# Indicators of mitochondrial disease

**DOI:** 10.1590/S1516-31802013000100012

**Published:** 2013-02-01

**Authors:** Yasemin Gulcan Kurt, Tuncer Cayci, Emin Ozgur Akgul, Erdinc Cakir

**Affiliations:** I MD. Assistant Professor, Department of Biochemistry, Gülhane Military Medical Academy, Ankara, Turkey.; II MD. Assistant Professor, Department of Biochemistry, Gülhane Military Medical Academy and Medical School, Ankara, Turkey.; III MD. Associate Professor, Department of Biochemistry, Gülhane Military Medical Academy and Medical School, Ankara, Turkey.

Dear Editor,

We read with great interest the article published by Silva et al. entitled “Steatosis of indeterminate cause in pediatric group: is it a primary mitochondrial hepatopathy?”.^1^ In the article, the authors hypothesize that hepatosteatosis with unknown etiology presented by group of pediatric patients may be mitochondrial disease. They attribute their idea to mitochondrial structural abnormalities detected through electron microscopy examination. They indicate that they have not found any significant difference in mitochondrial density between the control and other groups. To explain this situation, they suggest that the normal distribution of cytosol becomes altered because of the existence of lipid vacuoles in hepatocytes.

We would like to comment on their findings. First of all, in mitochondrial disease, mitochondrial proliferation almost always occurs because of mitochondrial dysfunction. Absence of increased mitochondrial density is not compatible with mitochondrial disease. Furthermore, the most reliable indicator of mitochondrial density is the level of citrate synthase activity in the tissue.^2^ Secondly, mitochondrial structural alterations do not provide strong enough evidence to indicate mitochondrial disease in the absence of other evidence, because many reasons other than mitochondrial disease can cause mitochondrial structural abnormalities.^3^^,^^4^ Thirdly, indicators for mitochondrial disease, such as lactic acid levels in blood, muscle histochemistry or mitochondrial enzyme activity were not evaluated in the article by Silva et al.^1^ At least mitochondrial deoxyribonucleic acid (mtDNA) alterations should be analyzed, because almost all mitochondrial disease involves mtDNA deletion, mtDNA depletion, mtDNA point mutation or nuclear deoxyribonucleic acid (nDNA) alterations.^5^ So, in our opinion, the evidence is not strong enough to hypothesize that this group of patients might have a mild form of primary mitochondrial hepatopathy.
