# Supplementary material for: Indicators of mitochondrial disease
Source: Sao Paulo Med J. 2013 Feb 1;131(1):64–5. doi: 10.1590/S1516-31802013000100012 (PMC10852078; doi:10.1590/S1516-31802013000100012)
Supplement: Related texts

# Reply: RESPONSE TO LETTER TO THE EDITOR

**DOI:** 10.1590/S1516-31802013000100012

**Authors:** Gustavo Henrique Silva, Gabriel Hessel, Kunie Iabiku Rabello Coelho, Cecília Amélia Fazzio Escanhoela

**Affiliations:** IV MD, PhD. Professor, Pharmaceutical Science College, Pontifícia Universidade Católica de Campinas (PUC-Campinas), Campinas, São Paulo, Brazil.; V MD, PhD. Assistant Professor, Department of Pediatrics, Faculdade de Ciências Médicas, Universidade Estadual de Campinas (FCM-Unicamp), Campinas, São Paulo, Brazil.; VI MD, PhD. Assistant Professor, Department of Pathology, Faculdade de Medicina de Botucatu, Universidade Estadual Paulista (FMB-Unesp), Botucatu, São Paulo, Brazil.; VII MD, PhD. Assistant Professor, Department of Pathology, Faculdade de Ciências Médicas, Universidade Estadual de Campinas (FCM-Unicamp), Campinas, São Paulo, Brazil.

We have carefully analyzed the remarks directed towards the original article “Steatosis of indeterminate cause in a pediatric group: is it a primary mitochondrial hepatopathy?”.^1^ Firstly, we would point out that the aim of our study was to evaluate and characterize “pure” steatosis in the pediatric group by means of morphological and morphometric analysis. This aim focused mainly on diagnostic characterization of the group of patients studied and not on elucidating the molecular mechanisms of the disease.

Some authors^2^^,^^3^^,^^4^^,^^5^ have taken the view that increased mitochondrial density, which may confer an oncocytic appearance on hepatocytes, is a major characteristic of microvesicular steatosis in non-alcoholic fatty liver disease (NAFLD). It is a possibility that increased mitochondrial density is an important causal factor of mitochondrial disease. Another indication of mitochondrial impairment is the frequent observation of megamitochondria, often showing crystalline inclusions. More recently, this has been indirectly correlated with oxidative stress in NAFLD.^6^

We agree that complementary analyses, such as determination of serum lactic acid and studies on mtDNA, make it possible to obtain more conclusive results. However, our study was retrospective, with material originating from biopsy files, which made it impossible to perform complementary analyses. Despite these limitations, we believe that the results support our conclusions, especially in relation to better characterization of mitochondrial morphological changes that are present in cases of fatty liver in children, for which causes such as metabolic diseases, obesity, undernutrition, infections and others were investigated and ruled out.

## References

[1] Silva GH, Hessel G, Coelho KI, Escanhoela CA (2011). Steatosis of indeterminate cause in a pediatric group: is it a primary mitochondrial hepatopathy?. Sao Paulo Med J.

[2] Sherlock S, Dooley J, Sherlock S, Dooley J (2002). Nutritional and metabolic liver diseases. Diseases of the liver and biliary system.

[3] Morris AA (1999). Mitochondrial respiratory chain disorders and the liver. Liver.

[4] Naviaux RK, Nyhan WL, Barshop BA (1999). Mitochondrial DNA polymerase gamma deficiency and mtDNA depletion in a child with Alpers' syndrome. Ann Neurol.

[5] Boustany RN, Aprille JR, Halperin J, Levy H, DeLong GR (1983). Mitochondrial cytochrome deficiency presenting as myopathy with hypotonia, external ophthalmoplegia, and lactic acidosis in an infant and as fatal hepatopathy in a second cousin. Ann Neurol.

[6] Sanyal AJ, Campbell-Sargent C, Mirshahi F (2001). Nonalcoholic steatohepatitis: association of insulin resistance and mitochondrial abnormalities. Gastroenterology.
